# Regulation of Calvarial Osteogenesis by Concomitant De-repression of GLI3 and Activation of IHH Targets

**DOI:** 10.3389/fphys.2017.01036

**Published:** 2017-12-19

**Authors:** Lotta K. Veistinen, Tuija Mustonen, Md. Rakibul Hasan, Maarit Takatalo, Yukiho Kobayashi, Dörthe A. Kesper, Andrea Vortkamp, David P. Rice

**Affiliations:** ^1^Orthodontics, Oral and Maxillofacial Diseases, University of Helsinki, Helsinki, Finland; ^2^Minerva Research Institute, Helsinki, Finland; ^3^Orthodontics, Tokyo Medical and Dental University, Tokyo, Japan; ^4^Center of Medical Biotechnology, University of Duisburg-Essen, Essen, Germany; ^5^Orthodontics, Oral and Maxillofacial Diseases, Helsinki University Hospital, Helsinki, Finland

**Keywords:** calvarial development, hedgehog signaling pathway, osteoblast, cell differentiation, craniosynostosis

## Abstract

Loss-of-function mutations in *GLI3* and *IHH* cause craniosynostosis and reduced osteogenesis, respectively. In this study, we show that *Ihh* ligand, the receptor *Ptch1* and *Gli* transcription factors are differentially expressed in embryonic mouse calvaria osteogenic condensations. We show that in both *Ihh*^−/−^ and *Gli3*^*Xt*−*J*/*Xt*−*J*^ embryonic mice, the normal gene expression architecture is lost and this results in disorganized calvarial bone development. RUNX2 is a master regulatory transcription factor controlling osteogenesis. In the absence of *Gli3*, RUNX2 isoform II and IHH are upregulated, and RUNX2 isoform I downregulated. This is consistent with the expanded and aberrant osteogenesis observed in *Gli3*^*Xt*−*J*/*Xt*−*J*^ mice, and consistent with *Runx2-I* expression by relatively immature osteoprogenitors. *Ihh*^−/−^ mice exhibited small calvarial bones and HH target genes, *Ptch1* and *Gli1*, were absent. This indicates that IHH is the functional HH ligand, and that it is not compensated by another HH ligand. To decipher the roles and potential interaction of Gli3 and Ihh, we generated *Ihh*^−/−^;*Gli3*^*Xt*−*J*/*Xt*−*J*^ compound mutant mice. Even in the absence of *Ihh, Gli3* deletion was sufficient to induce aberrant precocious ossification across the developing suture, indicating that the craniosynostosis phenotype of *Gli3*^*Xt*−*J*/*Xt*−*J*^ mice is not dependent on IHH ligand. Also, we found that Ihh was not required for *Runx2* expression as the expression of RUNX2 target genes was unaffected by deletion of *Ihh*. To test whether RUNX2 has a role upstream of IHH, we performed RUNX2 siRNA knock down experiments in WT calvarial osteoblasts and explants and found that *Ihh* expression is suppressed. Our results show that IHH is the functional HH ligand in the embryonic mouse calvaria osteogenic condensations, where it regulates the progression of osteoblastic differentiation. As GLI3 represses the expression of *Runx2-II* and *Ihh*, and also elevates the *Runx2-I* expression, and as IHH may be regulated by RUNX2 these results raise the possibility of a regulatory feedback circuit to control calvarial osteogenesis and suture patency. Taken together, RUNX2-controlled osteoblastic cell fate is regulated by IHH through concomitant inhibition of GLI3-repressor formation and activation of downstream targets.

## Introduction

The majority of the bones of the face and calvaria are formed by intramembranous ossification. Growth occurs primarily at the bone edges in the sutural joints (Rice and Rice, 2008). We and others have shown that across the suture there is the complete spectrum of osteogenic differentiation from quiescent stem cells through all osteoprogenitor stages to functioning mature osteoblasts. The expansion of each cell population is tightly regulated and dependent on the functional demands of the growing skull (Lana-Elola et al., 2007; Zhao et al., 2015; Maruyama et al., 2016). Osteoprogenitors condense at the osteogenic fronts and in order for osteogenesis to proceed a constant supply of progenitor cells must be available (Rice et al., 2003; Lana-Elola et al., 2007). Mis-regulation of the osteogenic condensations will result in alterations in skull bone shape and size, as well as in the patency of the sutures with consequent effects on skull growth. Within this niche, GLI transcription factors regulate stem cell maintenance, osteoprogenitor proliferation and differentiation (Rice et al., 2010; Veistinen et al., 2012). GLI1-positive cells in the suture mesenchyme are a source of mesenchymal stem cells, and ablation of *Gli1* in postnatal mice results in the premature fusion, or craniosynostosis, of all the calvarial sutures and the consequent secession of growth (Zhao et al., 2015). *GLI3* loss of function mutations cause craniosynostosis resulting in fusion across the interfrontal suture in patients and across the lambdoid and interfrontal sutures in mice (McDonald-McGinn et al., 2010; Rice et al., 2010; Hurst et al., 2011). These differences in phenotypes can, in part, be explained by location-specific differences in mRNA expression. *Gli1* is expressed evenly in all calvarial sutures while *Gli3* transcripts are more highly expressed in the interfrontal and lambdoid sutures compared to other calvarial sutures (Rice et al., 2010; Zhao et al., 2015).

GLI proteins are regulators of Hedgehog (HH) signaling, with GLI3 mainly functioning as a repressor of HH target genes. In the absence of the HH ligand GLI3 is proteolytically cleaved into the truncated repressor isoform, GLI3R (Wang et al., 2000). GLI3 acts as a strong repressor during limb patterning and during endochondral ossification, with SHH regulating limb patterning and IHH regulating cartilage development (Litingtung et al., 2002; Hilton et al., 2005; Koziel et al., 2005).

*Indian hedgehog* (*Ihh*) is expressed in the embryonic calvaria, and in osteoblastic cells has been shown to upregulate an osteogenic master regulator *Runx2* through GLI2 activator function (GLI2A) (Shimoyama et al., 2007; Rice et al., 2010). It has been proposed that IHH regulates several stages of osteoblast differentiation, either through Gli3R or Gli2A functions (Hilton et al., 2005; Joeng and Long, 2009). Although IHH is essential for osteoblast differentiation during endochondral ossification, it is not essential for intramembranous bone development. Osteoblasts appear to develop normally in calvarial bones of *Ihh*^−/−^ mice although the bones are reduced in size (St-Jacques et al., 1999; Lenton et al., 2011). By mapping the gene expression of *Ihh* in the developing chick dentary bone and analyzing the retroviral missexpression (gain of function) of *Ihh* in the developing chick frontal bone, it has been proposed that IHH regulates the transition of osteoprogenitors to osteoblasts. It is suggested that IHH does this by restricting the differentiation of preosteoblasts so that they can proliferate for longer which increases the pool of progenitors (Abzhanov et al., 2007). There is also evidence that IHH positively regulates osteoprogenitor recruitment to the osteogenic front possibly by controlling Bone morphogenetic protein (BMP) signaling (Lenton et al., 2011).

We have shown earlier how *Gli3*^*Xt*−*J*/*Xt*−*J*^ mice, which produce no functional GLI3 protein, can be used as a model to study craniosynostosis and suture biogenesis, and by genetically reducing the dosage of *Runx2* the calvarial phenotype can be rescued (Rice et al., 2010; Tanimoto et al., 2012; Veistinen et al., 2012). The transcription factor TWIST1 negatively regulates osteogenesis by inhibiting *Runx2* through direct DNA binding (Rice et al., 2000, 2010; Bialek et al., 2004), and in *Gli3*^*Xt*−*J*/*Xt*−*J*^ mice the expression of *Twist1* is reduced across the sutures. This aberrant signaling results in an increased pool of osteoprogenitors and enhanced osteogenic differentiation, with consequent ectopic bone formation and suture fusion. Support for this mechanism comes from premature activation of *Runx2* in embryonic day (E) 9.5 mice, as well as from the deletion of one allele of *Twist1* both of which result in enhanced osteogenesis and craniosynostosis (Bourgeois et al., 1998; Maeno et al., 2011). GLI3 repressor reduces RUNX2 activity by competing for the same DNA target sequences, thus negatively regulating osteoblast differentiation (Ohba et al., 2008; Lopez-Rios et al., 2012).

In this study we aimed to elucidate whether GLI3 controls IHH regulated intramembranous osteogenesis, and to test whether IHH, GLI3 and RUNX2 regulate and maintain the different stages of osteogenesis within the patent suture.

We show that IHH is the functional HH ligand in the embryonic mouse calvaria osteogenic condensations, where it regulates the progression of osteoblastic differentiation. We also demonstrate a location specific regulatory role for GLI3 repressor within the suture which is independent of IHH expression, as *Ihh* deletion does not rescue craniosynostosis exhibited by *Gli*3^*Xt*−*J*/*Xt*−*J*^ mice. In addition, we show that GLI3 represses the expression of *Runx2-II* and *Ihh*, and elevates *Runx2-I*. And that IHH is regulated by RUNX2 in calvarial osteoblasts. These data raise the possibility of a regulatory feedback circuit to control calvarial osteogenesis and suture patency.

## Material and methods

### Mice

The *Ihh*^+/−^ and *Gli3*^+/Xt−J^ mice maintenance, breeding and PCR genotyping has been described previously (Koziel et al., 2005). Wild-type (WT) littermates NMRI mice were used as controls. All animal experiments were approved by the University of Helsinki, Helsinki University Hospital and the Southern Finland Council Animal Welfare and Ethics committees.

### *In situ* hybridization

E13.5 and E15.5 tissues were fixed in a copious volume of freshly prepared 4% paraformaldehyde overnight at 4°C. They were then washed in PBS before dehydrating in an increasing strength ethanol or methanol series before *in situ* hybridization on either tissue sections or whole mounts, respectively.

*Ihh, Ptch1, Gli1-3, Runx2, Osx, Bglap*, and *Ibsp* riboprobes were prepared, and *in situ* hybridization was performed as described previously and briefly described below (Rice et al., 2006; Tanimoto et al., 2012).

### *In situ* hybridization on tissue sections

Sections (7μm) were deparaffinised, rehydrated and permeabilized with proteinase K. Tissues were hybridized overnight at 52°C with ^35^S-UTP labeled riboprobes. Hybridization was followed by high stringency washes at 50°C and at 65°C. Slides were then washed in NTE at 37° and treated with ribonuclease A to remove non-specifically bound and excess probe. The slides were coated with autoradiography liquid emulsion and exposed in a dark box for 10–18 days at 4°C. The slides were developed, fixed and then counterstained with hematoxylin.

### *In situ* hybridization on whole mounts

Samples were rehydrated, bleached with H_2_O_2_ and permeabilized with proteinase K. Tissues were prehybridized in PBST. Tissues were hybridized overnight at 64°C with denatured digoxigenin-labeled probes followed by high stringency washes. Next tissues were washed MABT then pre-blocked at room temperature for 3 h prior to overnight incubation with anti-dig-antibody coupled to alkaline phosphatase 4°C. Following extensive MABT and NTMT washes the color reaction was performed with NBT/BCIP.

### Immunohistochemistry

Immunohistochemical staining was performed as described previously (Rice et al., 2016). Briefly tissue sections were permeabilized and blocked, then incubated overnight at 4°C with anti-IHH (AF1705, R&D systems), anti-GLI1 (L42B10, Cell Signaling), anti-GLI3 (AF3690, R&D systems) or anti-PTCH1(G-19, Santa Cruz Biotechnology). Signal visualization was performed using Enzmet HRP detection (Nanoprobes). Sections were counterstained with nuclear fast red.

### Skeletal staining

Heads of mutant mice and WT littermates aged E16.5 and E18.5 were fixed in 95% ethanol overnight and stained with Alcian blue and Alizarin red and then cleared in 1% KOH and transferred to glycerol. For calculations of calvarial bone and suture size, images were captured using AnalySIS software (Soft Imaging System) and Olympus BX41 microscope and analyzed in Adobe® Photoshop CS4.

### Western blot analysis

Calvaria were dissected from E15.5 WT embryos and tissue samples taken from osteogenic front of the frontal bone and from interfrontal midsutural mesenchyme. Samples were pooled from three calvaria of the same litter and this was repeated 4 times. Age-matched brain tissue was used as control. Western blotting was carried out as described previously (Tanimoto et al., 2012). Briefly, 10 μg of each sample was probed for GLI3 antibody (AF3690, R&D Systems), GLI1 antibody (2643, Cell Signaling Technology), α-tubulin (T6199, Sigma-Aldrich). 20 μg of total protein from siRNA treated calvarial cells was probed for RUNX2 antibody (8486, Cell Signaling Technology), IHH antibody (AF1705, R&D Systems), and normalized against signal by Actin antibody (A2066 Sigma), detected by secondary antibodies goat anti-mouse IRDye 800CW (926-32210, LI-COR) and goat anti-rabbit 680RD (926-68071, LI-COR). Blots were analyzed with an Odyssey CLx infrared imager (model 9120) (Li-Cor) and Image-J (NIH) software. Statistical values were calculated using Student's *t-*test, with *P*-value below 0.05 indicated as significant.

### The effect of exogenous IHH on primary calvaria derived cells

Mouse E15.5 wild type (WT) primary calvaria derived cells (CDC) were isolated by trysinization and cultured for several days in DMEM containing high glucose and supplemented with 10% FBS. Cells were pooled from at least 2 calvaria. After 1st passage cells were seeded onto 6-well plates for experiments (100,000 cells per well). After 48 h of culture, cells were treated with recombinant human/mouse IHH protein (1705-HH-025 R&D systems): 50, 100, 250 ng/ml, and control cells with BSA (bovine serum albumin) for 1, 2, 6, and 24 h. RNA isolated from control and IHH treated CDC cells was quantified. 300 ng of RNA was used for cDNA synthesis using reverse transcriptase enzyme and random hexamer primers. Thereafter, RT-qPCR was performed using the primers *Gli1* (FP: CAGCATGGGAACAGAAGGACT, RP: CTCTGGCTGCTCCATAACCC), *Gli2* (FP: AACTTTTGTCTCCTCGGGTCC, RP: CTGCTGTCCTCCAAGAGACC) and *Gli3* (FP: AAGCCCATGACATCTCAGCC, RP: CTCGAGCCCACTGTTGGAAT). SYBR fast (Kapa Biosystems) qPCR master mix was used for real time quantitative PCR using Quant studio 3 from Thermo scientific. Mouse *18S rRNA* gene was used as the reference gene and negative control with no cDNA was used. The experiment conducted 3 times, each time with different biological samples.

### Calvarial osteoblasts and explant cultures

E15.5 mouse calvarial cells were isolated by four sequential trypsin-treatments of whole calvaria separated from the skin and meninges. The first set of cells after 15 min 0.25% trypsin incubation were discarded, and the cells from the following trypsin treatments were pooled and cultured in growth medium (DMEM containing 4,5 g/L glucose supplemented with 1 mM Na-pyruvate, 4 mM L-glutamine, 1% penicillin-streptomycine, and 10% FBS, (Lonza and Gibco).

Analysis of Wt and *Gli3*^*Xt*−*J*/*Xt*−*J*^ calvarial cells: At passage 2 the cells were transferred into new growth medium until confluent from which cell lysates were derived. Western blot analyses were carried out with 20 μg of total protein.

siRNA experiments: At passage 2 the cells were transfected either with negative control siRNA (Ambion Silencer Select control #1 siRNA, 4390843, Thermo Fisher) or *Runx2* siRNAs (Ambion 4390771 Runx2 Silencer Select Pre-designed siRNA, Thermo Fisher) using Lipofectamine RNAiMAX (Thermo Fisher). Cells were grown on six well plates with the siRNA transfection complex for 3 days, after which the medium was replaced by osteogenic medium (DMEM, 10 mM β-glycerophosphate, (Sigma), 50 μg/ml ascorbic acid, Sigma, and 100 ng/ml BMP2 (R&D Systems) for 24 h from which cell lysates were derived. Western blot analyses were carried out with 20 μg of total protein. siRNA experiments were carried out 3 times.

**Figure 1:**
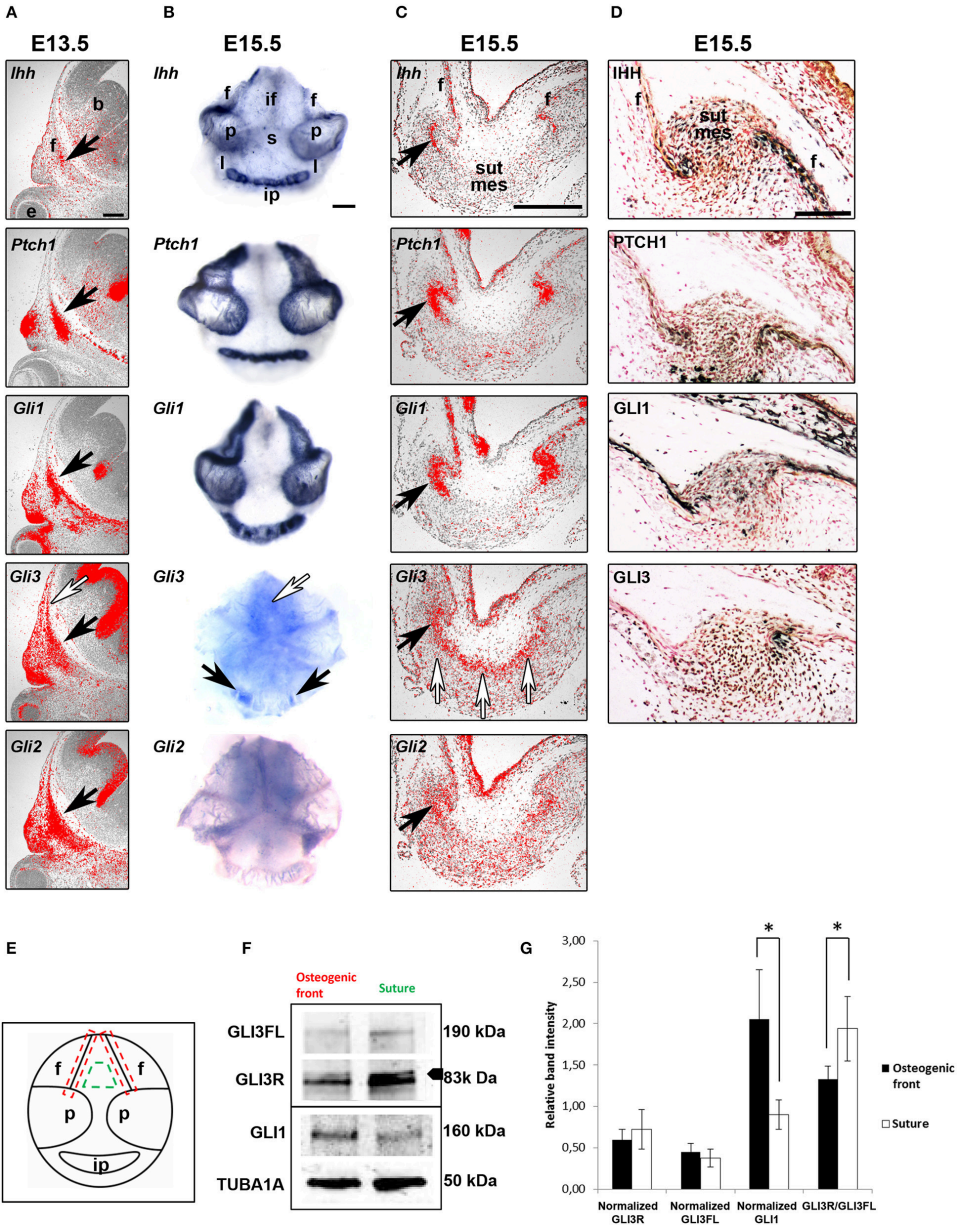
Regulatory role for GLI3R suggested by location-specific expression. **(A–C)** mRNA and protein expression **(D)** of hedgehog ligand, receptor and transcription factors in consecutive paracoronal calvarial tissue sections in E13.5 and E15.5 WT mice (black arrows). Ihh is expressed by a narrow strip of cells in the osteogenic fronts calvarial bone primordia. Ptch1, Gli1,-2 and−3 are also expressed in the osteogenic fronts but in a wider domain than Ihh. Gli3 is also expressed more distant from the osteogenic fronts in the midsutural mesenchyme (white arrows). **(E)** Schematic to show the areas of tissue sampling from E15.5 WT calvaria interfrontal midsutural mesenchyme (green) and osteogenic fronts of frontal bones (red). **(F)** Western blot analysis (triplicate), GLI3R (83 kDa) is the upper band indicated by the arrowhead. Integrated density values obtained for GLI3; both GLI3 full-length (GLI3FL) (190 kDa) and GLI3R, and GLI1 levels were normalized against those of α-tubulin and compared against the samples **(G)**. GLI3R/GLI3FL ratio is higher in the midsutural mesenchyme compared to the osteogenic front. Conversely, normalized GLI1 intensity is greater in the osteogenic fronts compared to the midsuture. Error bars standard deviation. Statistical values calculated using Student's *t-*test, with *p* < 0.05 considered significant (^*^p < 0.05). b, brain; e, eye; f, frontal bone; if, interfrontal suture, ip, interparietal bone; l, lambdoid suture; p, parietal bone; s, sagittal suture; sut mes, sutural mesenchyme. Scale bars: **(A–C)** 250 μm, **(D)** 100 μm.

E15 mouse calvarias were dissected for Trowell type of organ culture (Rice et al., 2000). Affi-gel agarose beads (Bio-Rad) were washed with PBS, and circa 200 beads were soaked with premixed siRNA—RNAiMAX reagent (Thermo Fisher) at 37°C for 30 min. Several beads were placed on the calvarias that were further cultured for 3 days in osteogenic medium. Efforts were made to place beads onto comparable areas of the calvaria that had endogenous expression of either *Runx2* full length or *Ihh* (Rice et al., 2010). The areas of *Ihh* expression in WT E15.5 calvaria were further identified (Figure 1B) and beads impregnated with either negative control or anti-RUNX2 SiRNAs were placed on the parietal bone osteogenic fronts for IHH experiments, which can be identified in bright field microscopy (Kim et al., 1998). Beads placed on the parietal bone or osteogenic fronts for RUNX2 experiments.

## Results

### Location specific regulatory role of GLI3 repressor

To determine the specific transcript (Figures 1A–C) and protein (Figure 1D) expression domains and protein activity of HH pathway members in the developing calvaria *in situ* hybridization and immunohistochemistry were performed. Detailed *in situ* hybridization was performed at different developmental stages of calvarial and suture maturation. In an attempt to get comparable data, at both E13.5 and E15.5 analysis was done on consecutive E15.5 calvarial tissue sections. Also, at E15.5 whole calvarial tissue was analyzed to determine whether all sutures have similar expression patterns.

*Ihh* mRNA and protein were detected in the osteogenic fronts of all the calvarial bones, in a very restricted group of osteoprogenitors (Figures 1C,D). In addition there was weak protein expression across the suture. The cell surface HH receptor *Ptch1* and *Gli1* and *Gli2* expression domains were overlapping with that of *Ihh*. *Ptch1* and *Gli1* are induced by HH ligand and therefore indicate the limit of Hedgehog activation (Lee et al., 2016). In contrast, *Gli3* was diffusely expressed across the sutural mesenchyme.

In general, the calvarial osteogenic fronts (E15.5) have similar expression patterns to the osteogenic condensations (E13.5). Indeed, the osteogenic fronts can be considered analogous to osteogenic condensations with similar steps and regulation of osteoblastic differentiation and development. We also observe a differential expression of *Gli3* in different sutures which may, in part, explain the suture specific phenotype seen in mice and patients with *GLI3* mutations.

To establish if GLI3R is the predominant protein isoform in the calvarial suture, we performed western blotting on tissue samples dissected from either the midsuture or the osteogenic front from WT calvaria (Figure 1E). The total amount of GLI3 protein was higher in the midsutural samples. Both isoforms were observed in both samples, but the level of GLI3R was higher in the suture compared to the osteogenic front, and the opposite was true for the GLI3FL (Full length) (Figures 1F,G). The ratio of GLI3R/GLI3FL was significantly higher in the suture mesenchyme indicating that GLI3R is predominate in the midsutural tissue.

To verify whether a sample was midsutural or from the osteogenic front, we blotted the samples against GLI1. GLI1 also acts as a read-out for HH signaling, and based on our *in situ* hybridization results, HH signaling is active only in the osteogenic front. The level of GLI1 protein was significantly higher in the osteogenic front samples compared to the samples isolated from the suture (Figures 1F,G). Taken together, HH signaling is activated in the osteogenic fronts and GLI3R is predominantly localized in the midsutural mesenchyme.

### GLI3 represses the expression of Runx2-II and Ihh, and elevates Runx2-I

**Figure 2:**
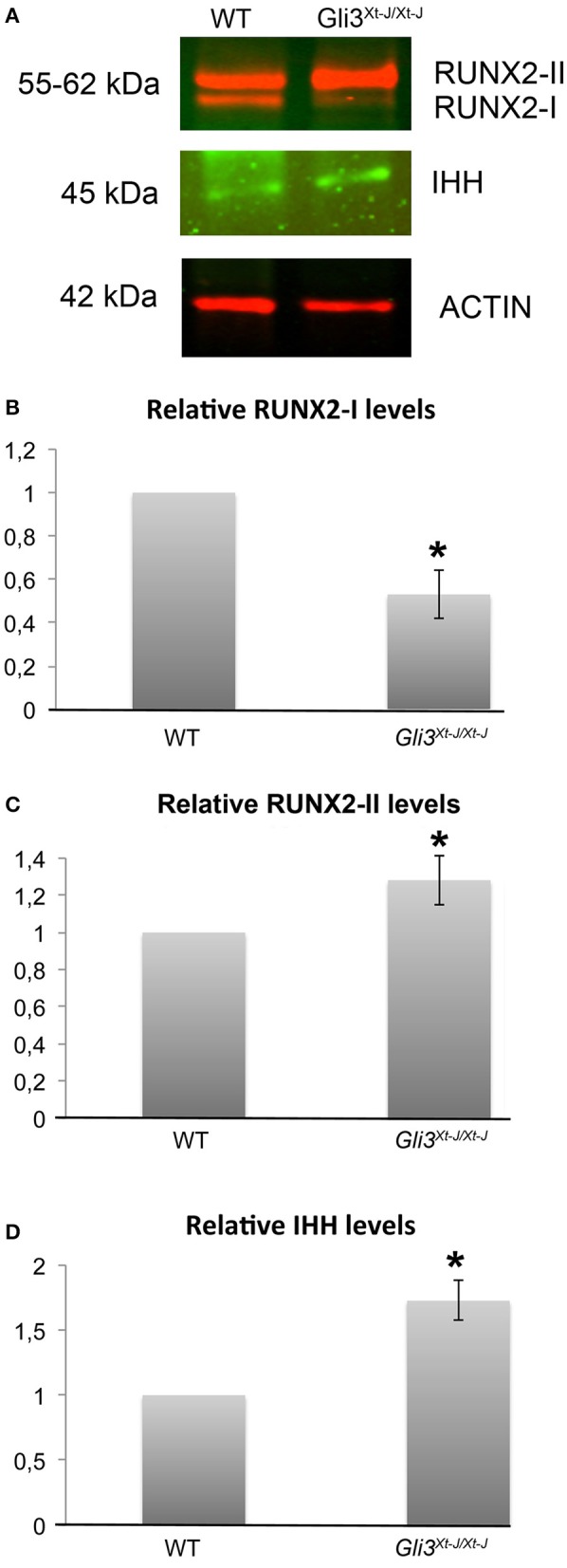
In the absence of *Gli3*, total RUNX2, RUNX2-II and IHH are upregulated and RUNX2-I downregulated. **(A)** Protein samples from *Gli3*^*Xt*−*J*/*Xt*−*J*^ calvarial osteoblasts in growth medium without osteogenic supplements show upregulated levels of RUNX2-II and IHH, and downregulated RUNX2-I, for 3 biological samples in each test group by six separate western blot analysis, representative results are shown. **(B,C)** Results are normalized against Actin antibody, and normalized values are shown with 1 representing the WT osteoblasts. (*p < 0.05) Error bars standard deviation.

RUNX2-I and RUNX2-II isoforms differ in their N-termini due to alternative promoter usage which results in dissimilar functions during bone development (Fujiwara et al., 1999; Zhang et al., 2009). Across developing sutures, *Runx2* isoforms are differentially expressed with *Runx2-I* primarily expressed in relatively undifferentiated osteoprogenitors and *Runx2-II* in cells further along in osteoblastic differentiation (Park et al., 2001). We have previously shown that *Runx2* is ectopically expressed across the *Gli3*^*Xt*−*J*/*Xt*−*J*^ suture (Rice et al., 2010). Here we analyzed their protein levels in osteogenic cells isolated from *Gli3*^*Xt*−*J*/*Xt*−*J*^ calvaria. We found that RUNX2-II was upregulated and RUNX2-I downregulated, compared to wild type controls indicating that in *Gli3*^*Xt*−*J*/*Xt*−*J*^ mice the osteoprogenitors within the suture were more differentiated than their WT littermates (Figures 2B,C). These results indicate that GLI3 maintains osteoprogenitors in an undifferentiated state by participating in the regulation of RUNX2. These data help explain the location specific differences seen in mRNA expression in *Gli3*^*Xt*−*J*/*Xt*−*J*^ calvaria and the craniosynostosis observed.

As *Runx2* expression is altered in *Gli3*^*Xt*−*J*/*Xt*−*J*^ calvaria (Figure 2) (Rice et al., 2010), and RUNX2 is known to regulate limb growth through the induction of *Ihh* (Yoshida et al., 2004), we analyzed IHH protein levels in *Gli3*^*Xt*−*J*/*Xt*−*J*^ cells. We found IHH to be upregulated (Figure 2D). This indicates that the lack of GLI3 has allowed either increased IHH expression or a broader IHH protein expression domain.

### Ihh does not alter the expression of *Gli3* mRNA

In mesenchymal cells of the developing limb bud, Sonic hedgehog is known to up-regulate *Gli* transcription, while down-regulating *Gli3* expression (Marigo et al., 1996). To test whether IHH has a direct effect on the expression of *Gli3* in WT primary calvaria derived cells (CDC) we added recombinant mouse IHH, of varying concentration, to mouse E15.5 calvarial cells and investigated *Gli1*, −2 and −3 expression levels RT-qPCR at different time points.

Exogenous IHH did not alter the mRNA levels of *Gli3* or *Gli2* (Supplementary Figure 1). At 24 h, IHH exposure resulted in a gradual up-regulation of *Gli1* expression in a dose-dependent manner (not statistically significant) (Supplementary Figure 1D). As *Gli1* is a known target of hedgehog signaling activation this response was used as a positive control. This is consistent with previous work in a chondrogenesis model using human bone marrow stromal/stem cells (Handorf et al., 2015).

### Ihh deletion does not rescue the *Gli3^*Xt*−*J*/*Xt*−*J*^* craniosynostosis

**Figure 3:**
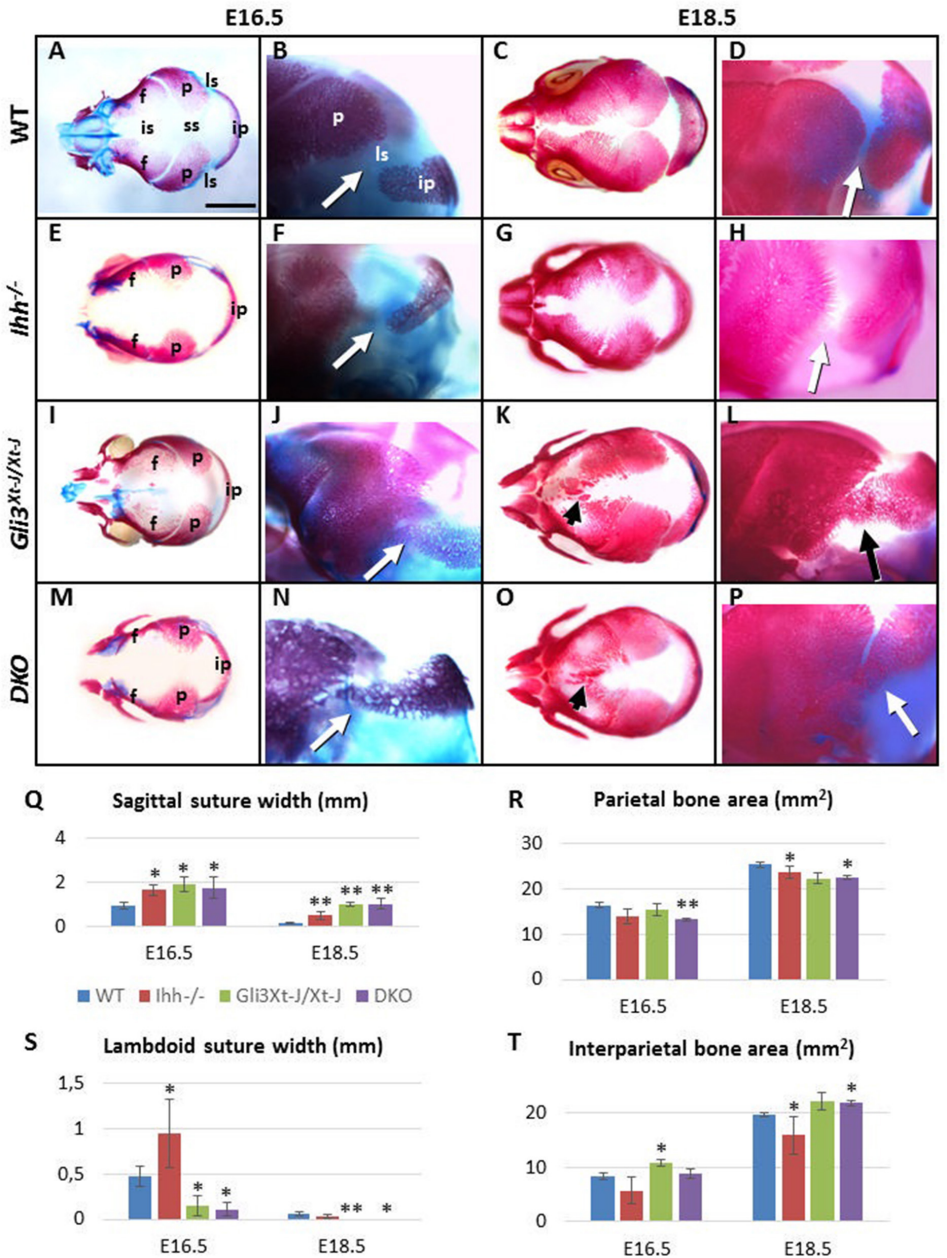
Craniosynostosis of *Gli3*^*Xt*−*J*/*Xt*−*J*^ mice is IHH ligand independent. **(A–P)** Alizarin Red-S and Alcian blue stained WT and mutant embryos. **(Q–T)** Selected suture width and bone area measurements. Compared to WT the calvarial bones of *Ihh*^−/−^ mice (*n* = 8) are smaller and sutures are wider compared (to the WT *n* = 20). *Gli3*^*Xt*−*J*/*Xt*−*J*^ mice (*n* = 24) have heterotopic ossifications in the interfrontal suture. The interparietal bone is larger and the lambdoid sutures are narrower with some samples already fused at E16.5. In contrast the sagittal suture is wider compared WT. Although the calvarial bone areas are reduced at E16.5, deletion of *Ihh* in *Ihh*^−/−^;*Gli3*^*Xt*−*J*/*Xt*−*J*^ (DKO) (*n* = 6) mice does not prevent the lambdoid sutures fusing (arrows) and heterotopic bones forming in the interfrontal suture (arrowheads) by E18.5. f, frontal bone; ip, interparietal bone; is, interfrontal suture; ls, lambdoid suture; p, parietal bone; ss, sagittal suture; DKO, double knockout (*Ihh*^−/−^;*Gli3*^*Xt*−*J*/*Xt*−*J*^). (^*^, *p* < 0.1, ^**^, *p* < 0.01 compared to WT). Error bars standard deviation. Scale bar 2 mm.

GLI transcription factors are effectors of HH signaling, with GLI3 acting as a strong repressor of IHH signals in chondrocyte differentiation with the developmental defects in endochondral ossification observed in *Ihh*^−/^^−^ mice being partially normalized by the loss of *Gli3 (Ihh*^−/−^;*Gli3*^*Xt*−*J*/*Xt*−*J*^ mice) (Koziel et al., 2005). We considered an analogous role during osteoblast development in the calvaria, and compared the calvarial phenotypes of *Ihh*^−/−^, *Gli3*^*Xt*−*J*/*Xt*−*J*^ and *Ihh*^−/−^*;Gli3*^*Xt*−*J*/*Xt*−*J*^ mutant mice, E16.5–18.5, to those of WT littermates (Figure 3).

Calvarial bones of *Ihh*^−/−^ mice were smaller than WT. The size of the frontal and the interparietal bones was most affected, the difference being the greatest at E16.5 [frontal bone 43% smaller (*p* < 0.05), interparietal bone 33% smaller compared to WT]. Concomitant with the reduced bone size was a widening of all of the calvarial sutures [E16.5: posterior interfrontal suture 32% wider (*p* < 0.05), sagittal suture 43% wider, lambdoid suture 45% wider. E18.5: lambdoid suture comparable to WT, posterior interfrontal suture 47% wider (*p* < 0.05), sagittal suture 71% wider (*p* < 0.05)] (Figures 3E–H,Q–T). The posterior part of the frontal bone was reduced in height (in apical direction) and the interfrontal suture was the widest posteriorly. At E18.5, the overall length of *Ihh*^−/^^−^ skull (nasal bones to the occiput) was slightly reduced (7%, not significant). This was in part due to the short cranial base (12% shorter, *p* < 0.05) (Figures 3E,G).

*Gli3*^*Xt*−*J*/*Xt*−*J*^ mutant mice showed very specific stage and location differences in bone size and suture width (Figures 3I–L,Q–T). Already at E16.5 25% of *Gli3*^*Xt*−*J*/*Xt*−*J*^ mice showed synostosis of the lambdoid suture. This was primarily due to an increase in the size of the interparietal bone which was enlarged from E16.5 onwards (Figure 3T) as opposed to a generalized increase in the size of the parietal bone. At E16.5 the width of the posterior interfrontal suture in *Gli3*^*Xt*−*J*/*Xt*−*J*^ calvaria was significantly wider (26%, *p* < 0.05). However, the surface area of the frontal bones was only 15% smaller. This mismatch of the widened interfrontal suture in between the near normally sized frontal bones, indicated that frontal bone development was affected from an early stage and that extrinsic factors, for example brain shape and size and may affect frontal bone and interfrontal suture morphology. Later, the size of the frontal bones relative to WT increased. This was in part due to strong heterotopic ossification in the interfrontal suture, which culminated in partial fusion of the suture at E18.5. (Figure 3K). At every stage examined the width of sagittal suture was significantly wider (*p* < 0.05) (51% wider compared to WT at E16.5, 87% wider at E18.5), but the size of parietal bones was not significantly smaller compared to WT (15% smaller at E16.5, 12% smaller at E18.5). This sagittal suture widening may be caused also by extrinsic factors. At E16.5 and E18.5 overall length of *Gli3*^*Xt*−*J*/*Xt*−*J*^ mutant skull was comparable to WT samples (Figures 3I–L).

In conclusion, *Gli3*^*Xt*−*J*/*Xt*−*J*^ calvaria exhibited enlargement of the interparietal bone and heterotopic interfrontal bones with consequent interfrontal and lambdoid suture fusion. In contrast, the same sutures were wider and the bones smaller in *Ihh*^−/^^−^ mice. These opposite differences led us to test whether *Gli3*^*Xt*−*J*/*Xt*−*J*^ phenotype could be rescued by deleting *Ihh*. The craniosynostosis phenotype across the interfrontal and lambdoid sutures in *Gli3*^*Xt*−*J*/*Xt*−*J*^ mice was unaffected by the loss of *Ihh* (Figures 3M–P), despite there being a small normalization of interparietal size at E16.5 (not significant) (Figure 3T). Thus, even in the absence of IHH ligand, *Gli3* deletion is sufficient to drive precocious ossification of the suture mesenchyme.

### Unlike *Ihh^−/−^* endochondral bones, osteoblastogenesis in *Ihh^−/−^* calvaria progresses normally

**Figure 4:**
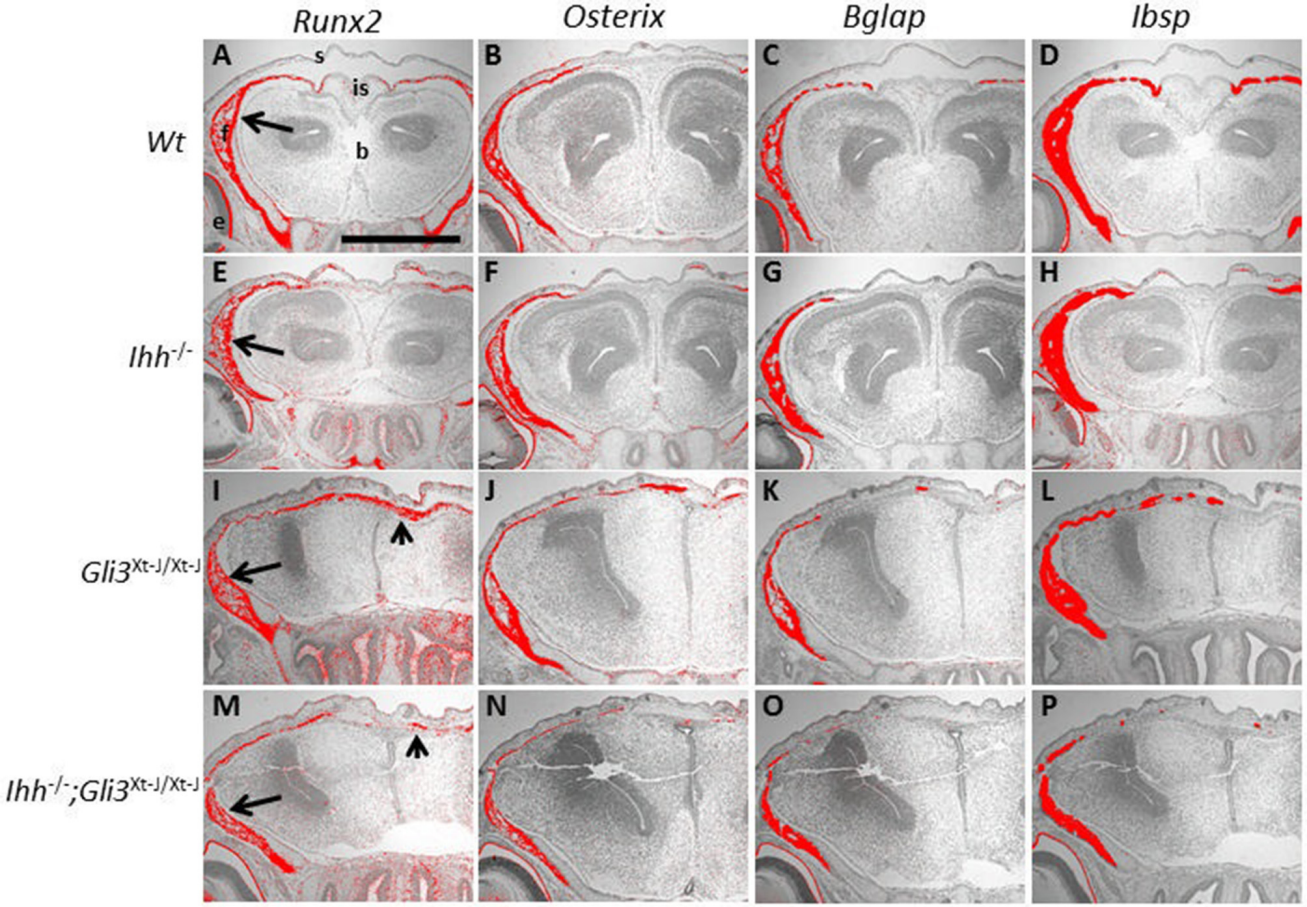
Osteoblasts differentiate in the absence of *Ihh* and *Gli3*. **(A–P)** E15.5 Frontal sections. *Runx2, Osterix* (*Osx*), *Bglap* (*Osteocalcin*) and *Integrin binding sialoprotein* (*Ibsp*) are all expressed in the WT frontal bones, shown by *in situ* hybridization (**A–D** arrows). All markers are also expressed in the *Ihh*^−/−^ frontal bones, but the interfrontal suture is wider **(E–H)**. In *Gli3*^*Xt*−*J*/*Xt*−*J*^ **(I–L)** and *Ihh*^−/−^;*Gli3*^*Xt*−*J*/*Xt*−*J*^ **(M–P)** calvaria *Runx2, Osx, Bglap* and *Ibsp* are expressed in the frontal bones, but expression also extends to the interfrontal suture (arrowheads). b, brain; e, eye; f, frontal bone; is, interfrontal suture; s, skin. Scale bar 500 μm.

*Ihh*^−/^^−^ mice exhibit a severe disruption of endochondral osteogenesis with a complete lack osteoblasts. In contrast *Ihh*^−/^^−^ mice do form calvarial bones (St-Jacques et al., 1999). Using *Runx2, Osterix* (*Osx, Sp7*), *Integrin binding sialoprotein* (*Ibsp*), and *Bglap* (*Osteocalcin)* as markers of increasing osteoblastic maturity, we analyzed osteoblastogenesis in *Ihh*^−/^^−^ embryonic calvaria (Figure 4). We found that osteoblastogenesis progressed normally even though the calvarial bones were small and thin, and the sutures wide with marker expression reflecting this phenotype (Figures 4E–H).

### De-repression of GLI3 in *Ihh^−/−^* mice results in a similar calvarial osteoblastic phenotype to *Gli3^*Xt*−*J*/*Xt*−*J*^* mice

In *Ihh*^−/^^−^ endochondral bones, we have previously shown that, de-repression of GLI3R (*Ihh*^−/−^;*Gli3*^*Xt*−*J*/*Xt*−*J*^ mice) is sufficient to restore the RUNX2-positive osteoprogenitor population (Koziel et al., 2005) but the additional action of GLI2A is required to restore the progression of osteoblastogenesis to the RUNX2-positive/Osterix-positive cell stage (Hilton et al., 2005). In *Ihh*^−/−^;*Gli3*^*Xt*−*J*/*Xt*−*J*^ calvaria the osteoblastic expression pattern was similar to that in *Gli3*^*Xt*−*J*/*Xt*−*J*^ mice although the heterotopic ossifications were less extensive (Figures 4M–P). Interestingly *Gli3*^*Xt*−*J*/*Xt*−*J*^ mice, which exhibit heterotopic ossifications in the interfrontal suture, showed expression of immature osteoblasts (*Runx2* and *Osx*) extending across the full width of the suture and more mature osteoblastic expression markers (*Ibsp* and *Bglap*) were limited to isolated ossification islands (Figures 4I-L).

### Ihh is the functional HH ligand in late embryonic mouse calvaria

**Figure 5:**
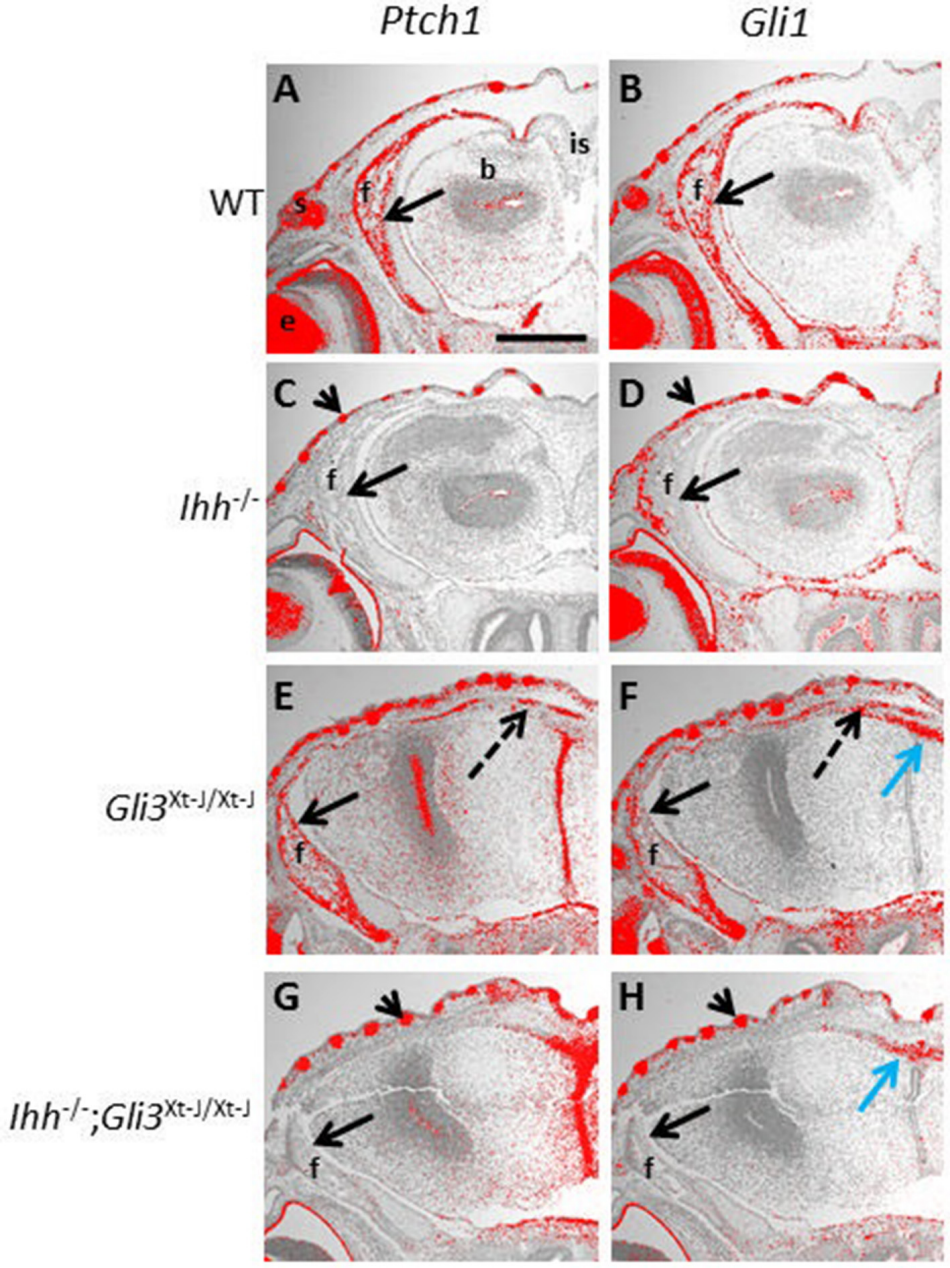
*Ptch1* and *Gli1* expression are not restored in *Gli3*^*Xt*−*J*/*Xt*−*J*^ calvaria by deletion of *Ihh*. **(A–H)** Hh target genes *Ptch1* and *Gli1* are upregulated by Hh ligand, E15.5 frontal sections are expressed in the developing WT frontal bone, shown by *in situ* hybridization (**A,B**, black arrows). However, in *Ihh*^−/−^ frontal bones *Ptch1* and *Gli1* are not expressed confirming that Ihh is the only Hh ligand expressed at this stage and that it activates *Ptch1* and *Gli1* (**C,D**, black arrows). In *Gli3*^*Xt*−*J*/*Xt*−*J*^ mice *Ptch1* and *Gli1* are expressed in the frontal bone (**E,F** black arrows), and in the interfrontal bones in the suture (**E,F** dotted black arrows). But in *Ihh*^−/−^;*Gli3*^*Xt*−*J*/*Xt*−*J*^ calvaria expression of both *Ptch1* and *Gli1* is absent (**G,H** black arrows). In *Ihh*^−/−^ and *Ihh*^−/−^;*Gli3*^*Xt*−*J*/*Xt*−*J*^ mice *Ptch1* and *Gli1* are expressed in the skin where they are activated by Shh (**C,D,G,H** arrowheads). In *Gli3*^*Xt*−*J*/*Xt*−*J*^ and *Ihh*^−/−^;*Gli3*^*Xt*−*J*/*Xt*−*J*^ mice *Gli1* is ectopically expressed in the apical midline of the malformed cerebrum, intracranial to the interfrontal suture where it is activated by Shh (**F,H** blue arrow). b, brain; e, eye; f, frontal bone; is, interfrontal suture; s, skin. Scale bar 1mm.

*Ptch1* and *Gli1* are direct transcriptional targets of HH signaling and both are upregulated by IHH (Lee et al., 2016). In *Gli3*^*Xt*−*J*/*Xt*−*J*^ mice, *Ptch1* and *Gli1* are expressed in the developing calvarial bones and in the abnormal craniosynostotic sutures. In *Ihh*^−/^^−^ calvaria however, *Ptch1* and *Gli1* were absent, indicating that no other Hh ligands are functional to compensate for the lack of Ihh (Figures 5A–F).

Endochondral ossification defects observed in *Ihh*^−/^^−^ mice can be partially normalized by deletion of *Gli3 (Ihh*^−/−^;*Gli3*^*Xt*−*J*/*Xt*−*J*^ mice). This rescue is accompanied by a concomitant normalization of *Ptch1* expression (Koziel et al., 2005). We therefore decided to test whether *Ptch1* and *Gli1* are normalized in the developing calvaria of *Ihh*^−/−^;*Gli3*^*Xt*−*J*/*Xt*−*J*^ mice. However, similar to *Ihh*^−/^^−^ calvaria, *Ptch1* and *Gli1* expression were absent in *Ihh*^−/−^;*Gli3*^*Xt*−*J*/*Xt*−*J*^ mice (Figures 5G,H). This suggests that the mechanism of *Ptch1* and HH signaling activation in the calvarial osteoprogenitors differs compared to that in chondrocytes in the developing growth plate.

### Ihh is regulated by runx2 in calvarial osteoblasts and calvarial explants

**Figure 6:**
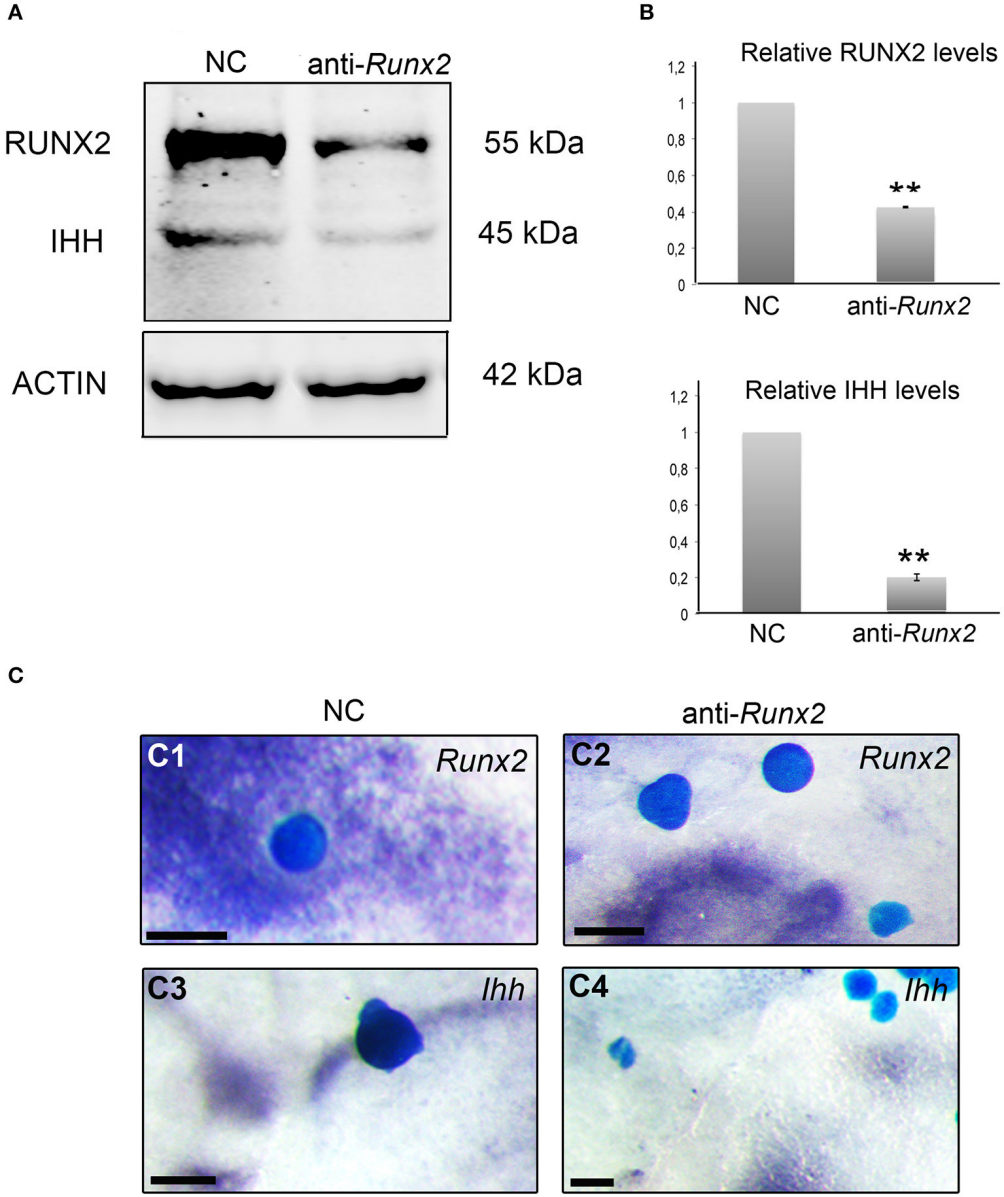
RUNX2 regulates the expression of IHH. **(A)** Protein lysates from negative control siRNA (NC) and *Runx2* siRNA treated calvarial osteoblasts analyzed by western blotting with anti-RUNX2 and anti-IHH antibodies (triplicate). *Runx2* siRNA downregulates RUNX2 and IHH. **(B)** Results are normalized against Actin antibody staining, and normalized values are compared and shown as columns with standard error, with 1 representing the WT osteoblasts. Statistical values calculated using Student's *t-*test, with *p* < 0.001 considered highly significant (^**^, *p* < 0.001). **(C)** Mouse E15.5 calvaria cultured with Affi-gel blue agarose beads soaked in negative control siRNA (NC) (*n* = 6) and anti-*Runx2* siRNA (*n* = 32), and subsequently analyzed by *Runx2* **(C1, C2)** and *Ihh* **(C3, C4)** *in situ* hybridization, with 28 out of 32 beads showing a reduction of *Ihh* **(C4)**. Note *Runx2* and *Ihh* expression adjacent to the negative control (NC) beads **(C1, C3)** but no *Runx2* and *Ihh* expression adjacent to the anti-*Runx2* siRNA beads **(C2, C4)**. scale bar 100 μm.

In the developing growth plate, it has been shown that Runx2 directly regulates *Ihh* expression in chondrocytes (Yoshida et al., 2004). To test an analogous interaction during intramembranous osteogenesis, we silenced *Runx2* in E15.5 primary calvarial osteoblasts by selective *Runx2* siRNA. Silencing was verified by western blot analysis showing that Runx2 protein was downregulated (Figures 6A,B). Ihh protein levels were significantly (*p* < 0.001) downregulated with *Runx2* siRNA, when compared to negative controls (Figures 6A,B). The regulation of *Ihh* by *Runx2* was then tested in embryonic calvarial organ culture with reduced expression of *Ihh* observed adjacent to beads coated in *Runx2* siRNA, assayed by whole mount *in situ* hybridization (Figure 6C).

## Discussion

Suture biogenesis involves the balance between the promotion and suppression of osteogenesis. The correct location specificity of the positive and negative osteo-inductive signals is important so that aberrant bone formation does not take place. It is not only important that the size and shape of the bones are controlled and that ectopic bones do not form but also that sutures stay patent to allow for continued brain and craniofacial growth.

Here, we show how skull bone size and shape are tightly controlled by hedgehog signaling in maintaining the required balance of progenitor cells and functioning osteoblasts, with Gli3 having a pivotal role. Gli3R acts as an inhibitor of osteogenesis in a location specific manner by repressing the osteo-inductive key players, RUNX2-II and IHH. GLI3R can act to negatively regulate RUNX2 or the function of RUNX2 via a least three possible mechanisms: GLI3 inhibits RUNX2 by competitively binding to the BGLAP (osteocalcin) promoter (Ohba et al., 2008; Lopez-Rios et al., 2012); GLI3 inhibits BMP2-DLX5 activation of RUNX2; and GLI3 upregulates *Twist1* which directly represses *Runx2* (Bialek et al., 2004; Tanimoto et al., 2012). RUNX2-I is down regulated in *Gli3*^*Xt*−*J*/*Xt*−*J*^ sutures and RUNX2-II upregulated. This is consistent with the expression of *Runx2-I* by a more immature osteoprogenitor population than those cells that express *Runx2-II* (Park et al., 2001; Tanimoto et al., 2012).

The effects of *Ihh* deletion on endochondral ossification are dramatic with long bones lacking osteoblasts (St-Jacques et al., 1999). IHH promotes osteogenesis in the orthotopic bone collar of endochondral bones through simultaneous GLI3 suppression and GLI2 activation (Hilton et al., 2005; Joeng and Long, 2009). Genetic expression of a constitutive *Gli2* activator is insufficient to rescue the defective osteoblastic differentiation in *Ihh*^−/−^ mice. However, the additional removal of *Gli3* restores *Runx2* and *Osx* expression (Joeng and Long, 2009). The regulation of osteogenesis in the calvaria differs from that in long bones as *Ihh*^−/−^ calvaria express *Runx2* and *Osx* (Figures 4E,F). That said, IHH positively regulates intramembranous osteogenesis with *IHH* duplications resulting in an overexpression of IHH being associated with craniosynostosis, and *Ihh*^−/−^ mice exhibiting small calvarial bones with expanded sutures (Figures 3E–H) (Klopocki et al., 2011; Lenton et al., 2011). Despite us demonstrating that IHH is the functional ligand for HH regulated osteogenesis in the calvaria (Figures 5C,D), deletion of *Ihh* in *Gli3*^*Xt*−*J*/*Xt*−*J*^ calvaria was not sufficient to rescue the craniosynostosis (Figures 3M–P). Despite the lack of IHH targets (*Ptch1* and *Gli1*) in *Ihh*^−/−^;*Gli3*^*Xt*−*J*/*Xt*−*J*^ mice (Figures 5G,H). This highlights the repressive role of GLI3 and also that craniosynostosis is not just excessive osteogenesis but a patterning defect.

Analysis of *Ihh* zebrafish mutants suggests that Ihh is involved in osteoprogenitor recruitment and proliferation and ultimately the regulation of dermal bone outgrowth and shape (Huycke et al., 2012). Also, following local retroviral mis-expression of *Ihh* in the developing chick frontal bone a developmentally later role for Ihh has been suggested where it regulates the transition from preosteoblastic progenitors to osteoblasts (Abzhanov et al., 2007).

Our results do not exclude the possibility that GLI3 has other roles during calvarial development even prior to the establishment of sutures. Inactivation of the BMP responsive transcription factors *Msx1* and *Msx2* from neural crest cells from E9.5–E10.5 causes heterotopic ossification in the interfrontal suture due to an alteration in the fate of the early migrating neural crest cells (Roybal et al., 2010). A population of calvarial mesenchymal cells arise from HH-responsive *Gli1*-positive cells in the cephalic paraxial mesoderm and although this transcriptional activation of *Gli1* is transient, GLI3 may have a role in controlling migration and patterning of these cells (Deckelbaum et al., 2012).

During calvarial osteoblast differentiation *Gli3* is expressed prior to *Ihh* expression and then it functions as a repressor of HH target genes. During limb patterning the situation is similar, as GLI3 and dHand prepattern the limb bud before dHand activates SHH signaling (te Welscher et al., 2002). Evidence also shows that mouse limbs expressing only GLI3R have only one digit and thus resemble *Shh* null allele mice. The primary mechanism by which SHH patterns the anteriorposterior limb is by inhibiting the GLI3R formation (Cao et al., 2013).

Two mouse models of craniosynostosis have been reported with abnormal processing of GLI3 as an etiological factor. An ENU-induced mouse model representing a hypomorphic allele of *Ptch1* has bilateral craniosynostosis of the lambdoid suture, similar to *Gli3*^*Xt*−*J*/*Xt*−*J*^ mice (Feng et al., 2013). This phenotype is caused by a ligand-independent activation of HH signaling and a decrease in GLI3R. Fuzzy is a regulator of cilia trafficking and consequently HH signaling. Despite, *Fuzzy* null allele mice have increased repression of GLI3R they also exhibit craniosynostosis, all be it a different suture from that fused in *Gli3*^*Xt*−*J*/*Xt*−*J*^ and *Ptch1* mutant mice. The coronal suture fusion seen in *Fuz*^−/−^ mice is associated with elevated fibroblast growth factor signaling which is known to play a role in regulating suture patency (Tabler et al., 2013).

We found that in *Gli3*^*Xt*−*J*/*Xt*−*J*^ cells, RUNX2 and IHH are elevated and silencing *Runx2* decreases *Ihh* expression (Figures 6A,B). This suggests that RUNX2 may control *Ihh* expression during calvarial osteoblast differentiation, which would be analogous to its role during chondrogenesis (Yoshida et al., 2004). *Ihh* is expressed by progenitors in the osteogenic fronts (Figures 1A,B) and it signals to the cells within the osteogenic condensation to differentiate via a GLI2-driven mechanism. Proteolytic processing of GLI3 into the repressor isoform is inhibited in these cells allowing osteoblast differentiation to proceed. *Runx2* is upregulated and in turn activates *Ihh* expression (Figures 2A–C).

## Conclusions

**Figure 7:**
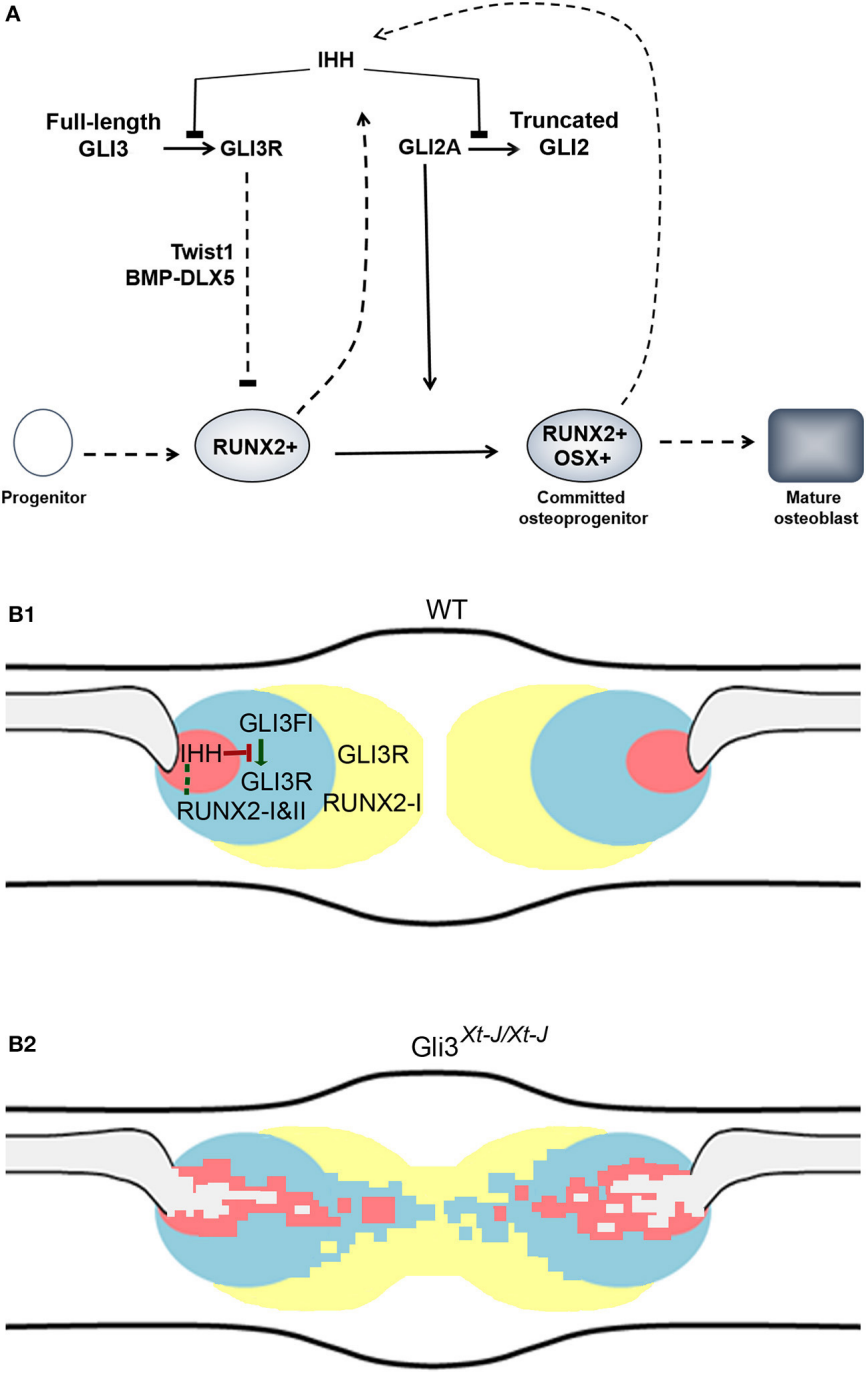
Tight regulation of calvarial osteogenesis by GLI3. **(A)** IHH inhibits the proteolytic truncation of GLI3 into its repressor form (Wang et al., 2000). GLI3R inhibits RUNX2 by competitively binding to the BGLAP (osteocalcin) promoter (Ohba et al., 2008; Lopez-Rios et al., 2012); GLI3R inhibits BMP2-DLX5 activation of RUNX2; and GLI3R upregulates *Twist1* which directly represses *Runx2* (Bialek et al., 2004; Tanimoto et al., 2012). IHH promotes GLI2A which permits the progression of osteoblast differentiation from RUNX2-positive to RUNX2-positive/OSX-positive progenitors (Shimoyama et al., 2007). Our data suggests that RUNX2 may also participate in the regulation IHH. Figure is adapted from (Joeng and Long, 2009), and (Long and Ornitz, 2013) (Joeng and Long, 2009; Long and Ornitz, 2013). **(B1)** Osteogenesis in the normal suture. GLI3 primarily acts as an inhibitor of intramembranous ossification at the periphery of the bones calvarial bones. The truncated repressor form of GLI3 (GLI3R) is the predominant isoform in the undifferentiated sutural mesenchyme (yellow) and at the peripheral edge of the osteogenic condensation (blue). GLI3R restricts ossification and limits the proliferation rate of the mesenchymal osteoprogenitor cells by upregulating *Twist1* **(1)**. In midsutural mesenchyme *Runx2-I* expression by relatively undifferentiated progenitors is high, while in the osteogenic condensation the *Runx2* promoter is activated and both *Runx2* isoforms are upregulated. *Ihh* expression is highly localized within the osteogenic condensation possibly resulting in a short range activation of target genes, including GLI2A. GLI3 repressor limits bone formation at the edge of the osteogenic condensation. In the osteogenic condensation RUNX2-I and RUNX2-II act in a feedback loop by upregulating IHH (dashed line). Within the Ihh signaling domain (red) the truncation of GLI3FL to GLI3R does not occur and osteogenesis is activated. Outside the IHH signaling domain, GLI3R is generated which inhibits osteogenesis and maintains suture patency. **(B2)** In *Gli3*^*Xt*−*J*/*Xt*−*J*^ calvaria, as GLI3R does not function, the tight regulation of osteogenesis is lost. This permits the expression domains of IHH and RUNX2 to expand and bone to form across the suture. Key: GLI3 Full length (GLI3FL), GLI3 repressor (GLI3R), undifferentiated sutural mesenchyme (yellow), osteogenic condensation periphery (blue), osteogenic condensation center (red), bone matrix (gray).

The stepwise differentiation of osteoblasts from mesenchymal progenitors through developmental stages characterized by the expression of SOX9, RUNX2, and OSX is well described (Long and Ornitz, 2013) (Figure 7A). IHH acts at 2 stages prior to and post Runx2 positive cells (Joeng and Long, 2009). IHH inhibits the post translational cleavage of GLI transcription factors into truncated repressor forms (Wang et al., 2000). Thus, when IHH is expressed GLI1 and −2 function as activators of downstream targets to regulate osteogenesis. GLI1 regulates calvarial stem cells while GLI2 acts an activator which, together with the simultaneous removal of GLI3R, allows the progression of uncommitted RUNX2-positive osteoprogenitors into committed RUNX2-positive/OSX-positive osteoprogenitors (Shimoyama et al., 2007; Joeng and Long, 2009; Zhao et al., 2015).

We and others have previously shown that GLI3R regulates calvarial osteogenesis by negatively regulating the action of RUNX2. With RUNX2, GLI3R competitively binds to the BGLAP (osteocalcin) promoter (Ohba et al., 2008; Lopez-Rios et al., 2012). GLI3R inhibits the Bmp2-Dlx5 activation of Runx2 and upregulates TWIST1 which directly represses RUNX2 (Bialek et al., 2004; Tanimoto et al., 2012).

We show that IHH is the functional HH ligand in the late embryonic mouse calvaria. But unlike Ihh^−/−^ endochondral bones, osteoblastogenesis in Ihh^−/−^ calvaria progresses normally. So IHH is not essential for calvarial osteoblastic differentiation (St-Jacques et al., 1999). As IHH regulates the conversion of full-length GLI3 into GLI3R, within the IHH signaling domain the truncation of GLI3FL to GLI3R does not occur and osteogenesis is activated. Outside the IHH signaling domain, in immature mesenchymal progenitor cells, GLI3R is generated which inhibits osteogenesis (Figure 7B). Here we demonstrate a location specific regulatory role for GLI3R within the suture which is independent of *Ihh* expression, as *IHH* deletion does not rescue craniosynostosis exhibited by Gli3^Xt−J/Xt−J^ mice. We show that GLI3 represses the expression of *Runx2-II* and *Ihh*, and elevates *Runx2-I*. And that IHH is regulated by RUNX2 in calvarial osteoblasts and calvarial explants. This raises the possibility of a regulatory feedback circuit to control calvarial osteogenesis and suture patency.

While we have found some interesting results with regard to intra-suture regulation of, particularly, the interfrontal suture, we have not made extensive analyses of different sutures and at multiple developmental stages. With these limitations in mind, caution is emphasized when extrapolating conclusions we make from data mainly from the interfrontal suture to other sutures.

Taken together, we have presented evidence how GLI3R, RUNX2 and IHH regulate the stage and location of osteoblast differentiation in the calvarial sutures, which ultimately controls skull bone patterning, shape and size.

## Author contributions

Study design: LV, TM, MH, MT, AV, and DR, Data collection, analysis, and interpretation: LV, TM, MH, MT, YK, DK, AV, and DR, Drafting manuscript and final version approval: LV, TM, MH, MT, YK, DK, AV, and DR, Responsibility for the integrity of the data analysis: LV, TM, MH, MT, YK, DK, AV, and DR.

### Conflict of interest statement

The authors declare that the research was conducted in the absence of any commercial or financial relationships that could be construed as a potential conflict of interest.

## Supplementary material

Supplementary Figure 1 IHH does not affect the expression of *Gli3* mRNA. **(A–D)** *Gli1*,−2 and −3 expression levels in WT primary calvaria derived cells analyzed by RT-qPCR at 1, 2, 6, and 24 h in response to application of 50, 100 and 250 ng/ml of recombinant mouse IHH. Exogenous IHH did not alter the mRNA levels of *Gli2* or *Gli3*. At 24 h, IHH exposure resulted in a gradual up-regulation of *Gli1* expression in a dose-dependent manner (not statistically significant). As *Gli1* is a known target of hedgehog signaling activation this response was seen as a positive control.
