# Supplementary figures and images for: Regulation of Calvarial Osteogenesis by Concomitant De-repression of GLI3 and Activation of IHH Targets
Source: Front Physiol. 2017 Dec 19;8:1036. doi: 10.3389/fphys.2017.01036 (PMC5742257; doi:10.3389/fphys.2017.01036)

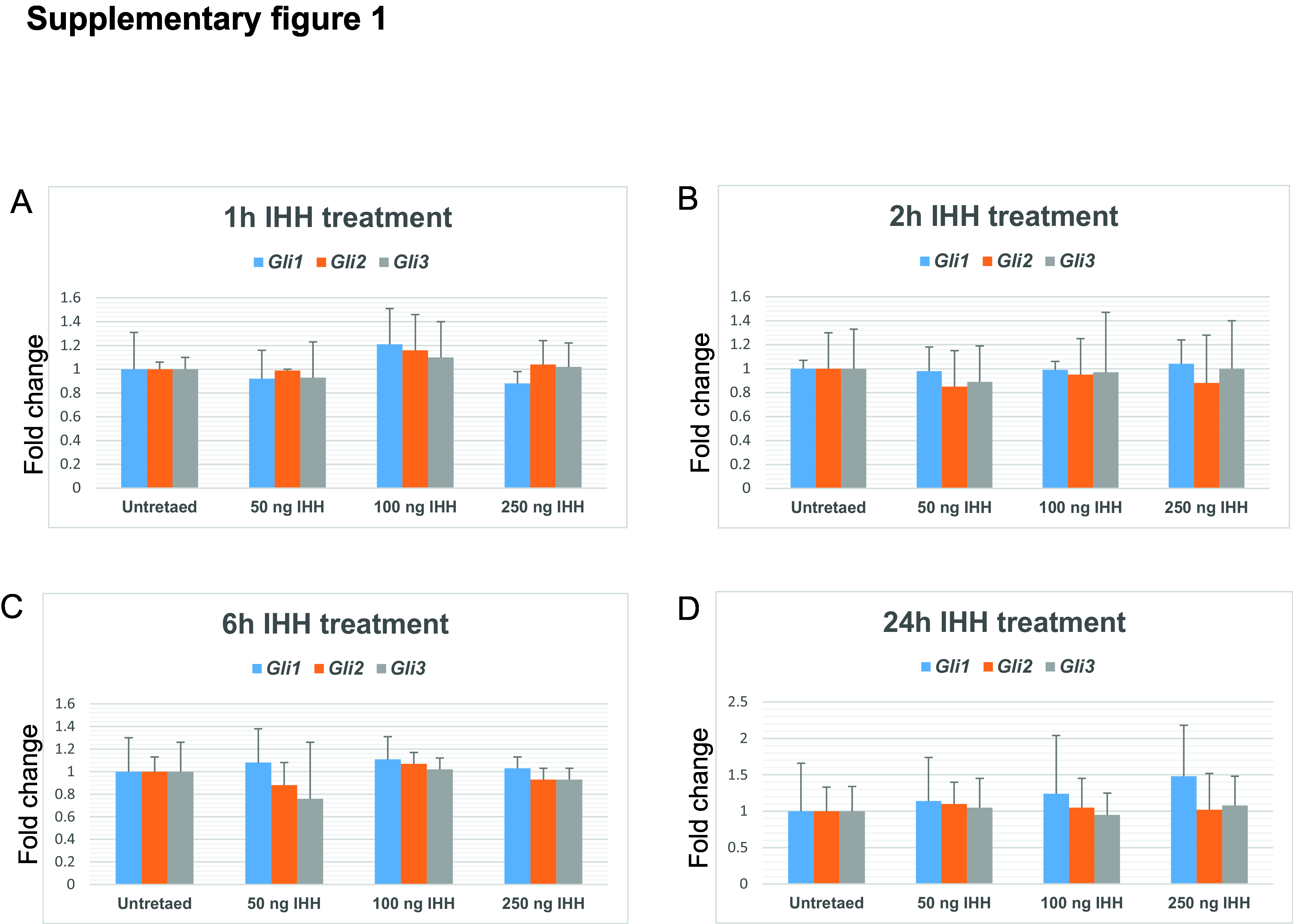

Supplement: Supplementary Figure 1 — IHH does not affect the expression of Gli3 mRNA. (A–D) Gli1,−2 and −3 expression levels in WT primary calvaria derived cells analyzed by RT-qPCR at 1, 2, 6, and 24 h in response to application of 50, 100 and 250 ng/ml of recombinant mouse IHH. Exogenous IHH did not alter the mRNA levels of Gli2 or Gli3. At 24 h, IHH exposure resulted in a gradual up-regulation of Gli1 expression in a dose-dependent manner (not statistically significant). As Gli1 is a known target of hedgehog signaling activation this response was seen as a positive control. [file Image1.JPEG]
